# The C-Terminal O-S Acyl Shift Pathway under Acidic Condition to Propose Peptide-Thioesters

**DOI:** 10.3390/molecules21111559

**Published:** 2016-11-17

**Authors:** Bo Mi Kim

**Affiliations:** Division of Bio-Nanochemistry, Wonkwang University, Iksan 570-749, Korea; 123456@wku.ac.kr; Tel.: +82-63-850-6230

**Keywords:** C-terminal handles, peptide thioesters, acid-catalyzed O-S acyl shift

## Abstract

Peptide-thioester is a pivotal intermediate for peptide ligation and N-, C-terminal cyclization. In this study, desired pathway and the side products of two C-terminal handles, hydroxyethylthiol (HET) and hydroxypropylthiol (HPT) are described in different conditions as well as kinetic studies. In addition, a new mechanism of C-terminal residue racemization is proposed on the basis of differentiation of products derived from the two C-terminal handles in preparing peptide thioesters through an acid-catalyzed tandem thiol switch, first by an intramolecular O-S acyl shift, and then by an intermolecular S-S exchange.

## 1. Introduction

**Scheme 1:**
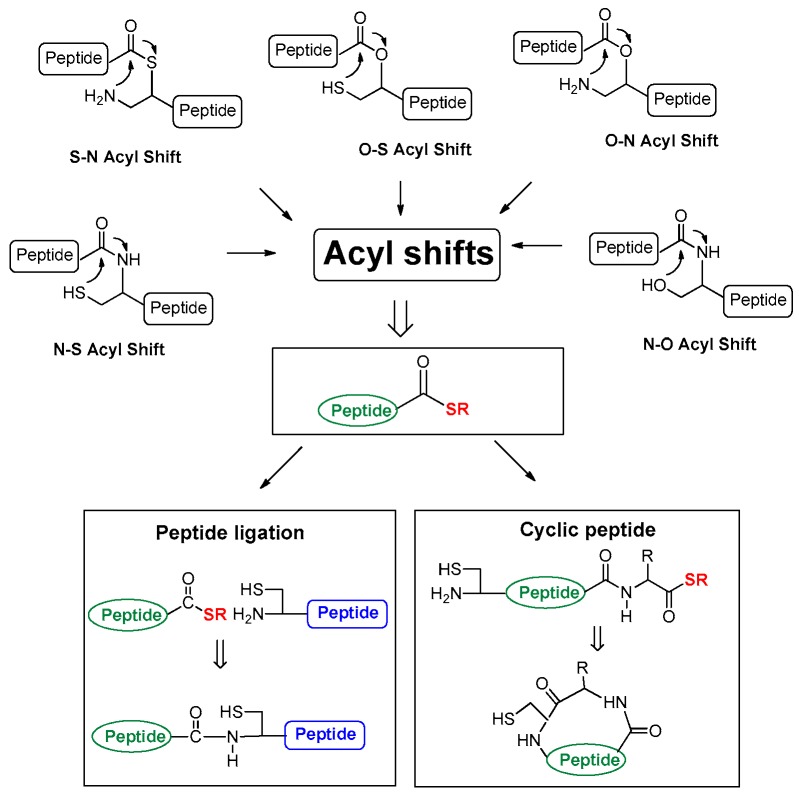
Acyl shifts involved in peptide manipulation.

Acyl migration, which is explained as acid- or base-catalyzed acyl shift has been discovered in peptide bonds and their alternate structures [1,2,3,4,5,6]. Although early studies have claimed whether the process produces complications [7] or utility [4], Kemp rendered a milestone example of acyl shift using a prior capture strategy [8] that presented a thiol capture in hexafluoro-2-propanol and water (4:1 ratio) and an intramolecular O-N acyl shift under a basic condition without a coupling reagent [8]. After Kemp’s example in 1981, the idea of acyl shift has been rapidly applied to peptide ligation methodology using various chemical [9,10,11,12,13,14,15,16,17,18,19,20,21,22,23,24,25,26] and enzymatic [27,28] methods, including intein-mediated protein splicing [29,30]. Many acyl shifts (Scheme 1) that contain S-N [10,11,12,13,14], N-S [17], O-N [16,17,18], N-O [19] and O-S [31,32,33,34,35,36,37,38,39,40,41,42] have been developed as a pivotal step of peptide manipulation under acidic or basic conditions.

**Scheme 2:**
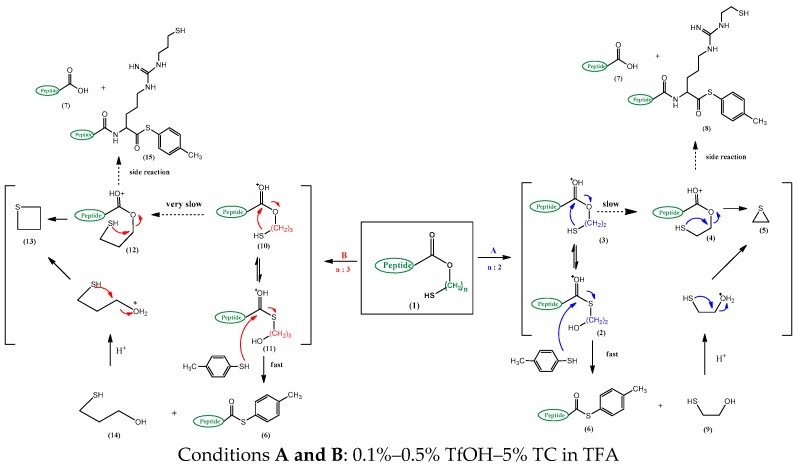
The product pathways of peptide Threonine-Isoleucine-Glycine-Glycine-Isoleucine-Arginine Hydroxyethylthiol(TIGGIR-HET) and peptide Threonine-Isoleucine-Glycine-Glycine-Isoleucine-Arginine Hydroxypropylthiol(TIGGIR-HPT) under acidic conditions.

Recent studies have described the preparation of peptide thioesters through an acid-catalyzed tandem thiol switch, first by an intramolecular O-S acyl shift, followed by an intermolecular S-S exchange, after attaching a hydroxyethylthiol (HET) handle, suitable for 9-fluorenyl methoxyl oxycarbonyl (Fmoc) chemistry on the commercially available 2-chlorotrityl chloride resin [43]. The peptide ester **1** (Scheme 2) permits an acid-catalyzed [trifluoromethanesulfonic acid (TfOH) in trifluoroacetic acid (TFA)] and an intramolecular O-S acyl shift **2**→**3** followed by an intermolecular S-S exchange **2**→**6** caused by an external thiocresol (TC) to produce the peptide thioester **6**.

**Scheme 3:**
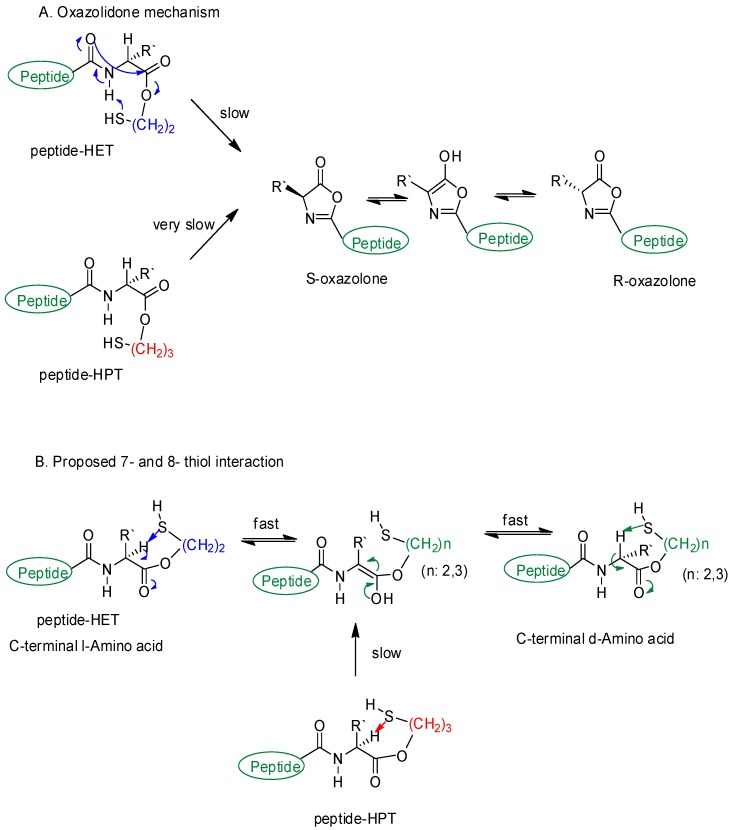
Proposed racemization pathways at C-terminal residue. (**A**) is oxazolidone mechanism and (**B**) is proposed mechanism.

However, a systematic approach for O-S acyl shift has not been fully established because the core structures for O-S acyl shift were susceptible to side reactions such as hydrolysis and C-terminal racemization [31,32,37,40], which have been found in both intermolecular [31,32,37] and intramolecular [33,34,35,36,38,39,40] methods using various metal-mediated [31,32], acidic [33,37,43], neutral [35], and basic [34,36,38,39,40,41,42] conditions. Similar with other studies [31,32,33,37,40] the two critical problems from TIGGIR-OEtSH (TR6-HET) **3** which were encountered, are the alkylation of **8**, the guanidino side chain of Arginine (Arg) by ethylene sulfide **5** (Scheme 2) and the C-terminal racemization (Scheme 3). Due to lack of reasonable approaches, it had been difficult to explain the side products pathway from an application-oriented design with only the HET handle structure. In the present study, the product pathway is demonstrated using HET and hydroxypropylthiol (HPT) handles as well as the kinetic studies, and a new mechanism of C-terminal racemization which was deduced from the results of the universal C-terminal handles in the preparation of TR6-thioester **6** is proposed.

## 2. Results and Discussion

**Figure 1:**
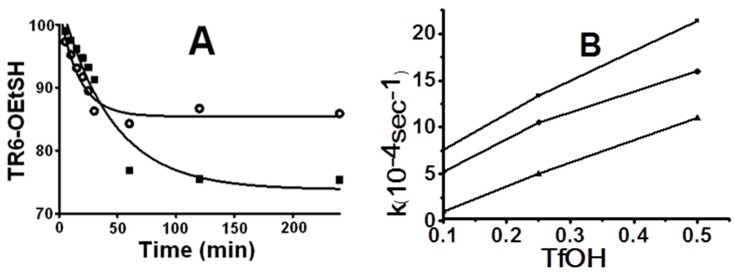
Time courses and rate effect on the basis of disappearance of TR6-HET and TR6-HPT at different conditions: (**A**) the time courses of peptide TIGGIR-HET disappearance in 5% TC-TFA (■) and TFA only (●); and (**B**) rate effect of the TfOH additions on disappearances of TIGGIR-HET in 5% TC-TFA (●), TIGGIR-HET in 5% TC-TFA (■), and TIGGIR-HPT in 5% TC-TFA (▲).

In the study for the effect of acidity in the reaction medium, a very important factor for O-S acyl shift is monitoring the TFA conditions without TfOH, such as 5% Thiocresol-TFA (TC-TFA) or TFA alone, by TR6-HET disappearance (Figure 1A). The conditions gave very slow disappearance rates, even though the 5% TC-TFA condition accelerated a little after 30 min. Furthermore, the main conversion product within a short period of time in both conditions (Figures S1 and S2) was the racemized TIGGIR-OEtSH (TR6-HET), which was confirmed through direct synthesis (Figure S5), while the 5% TC-TFA condition led to CF_3_CO-acetylated products after a long period (Figure S2). These results were consistent with previous findings [43] that the HET handle of peptide ester 1 requires an increase in acidity to protonate the ester carbonyl for the thioester conversion.

To examine the effect of further increase in acidity, the first-order rates of TR6-HET in TFA and 5% TC-TFA conditions were compared with 0.1%, 0.25%, and 0.5% TfOH (*v/v*) additions. The experiment gave rise to distinct rates of 1,2-elimination and O-S acyl shift, while the main products detected were peptide-TCs in TC-TfOH-TFA (Scheme 2A,B), peptide-TFA adducts in TfOH-TFA (Figure S2), and peptide-OHs in TFA only (Figure S1). The 1,2-elimination progressed relatively slowly, compared to the five-membered O-S acyl shift (Figure 1B). Furthermore, the S-S exchange seems to be a crucial step to isolate the stable thioester peptides; since otherwise the 1,2-elimination which produces the acid form, becomes more dominant than the unstable 5-membered O-S acyl shift. The catalytic TfOH additions into peptide-HET allowed a similar product profile with subtle differences in a broad range (0.1%–0.5%) instead of the expected narrow optimal point (Table S1).

**Figure 2:**
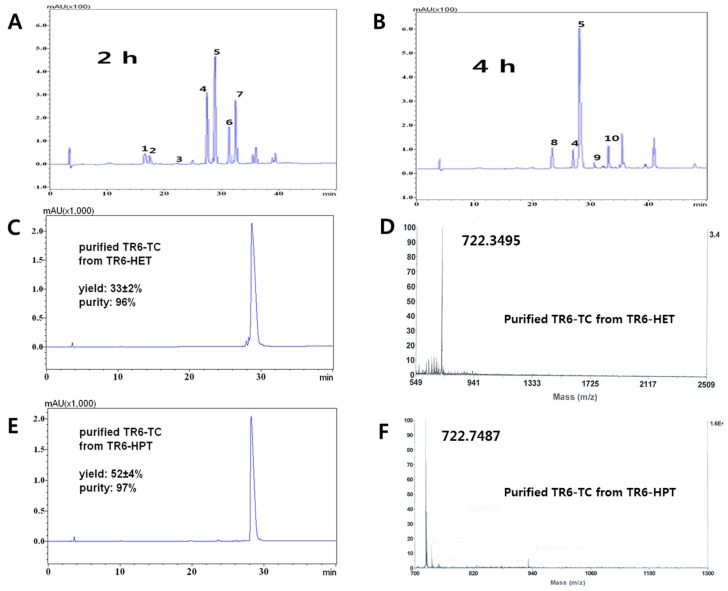
HPLC profiles of the main products between two different C-terminal handles, HET and HPT. Condition: 0.5% TfOH-5% TC-TFA (total volume; 1 mL): (**A**) TIGGIR-OEtSH (TR6-HET, Scheme 2, (3)), 2 h, 2–3 mg; and (**B**) TIGGIR-OPrSH (TR6-HPT, Scheme 2, (10)), 4 h, 2–3 mg. The peptides were precipitated by diethylether to remove TC and TfOH. **1**, **2**: TR6-OH and TR6-OH (*m*/*z* 616.4); **3**: TR6-HET (*m*/*z* 676.4); **4**: TR6-TC (*m*/*z* 722.4), which was confirmed by direct synthesis of TR6-HET (Figure S6); **5**: TR6-TC (*m*/*z* 722.4); **6**: TR(EtSH)6-TC (*m*/*z* 782.4); **7**: TR(EtSH)6-TC (*m*/*z* 782.4); **8**: TR6-HPT (*m*/*z* 690.4); **9**: TR(OPrSH)6-TC (*m*/*z* 796.4); **10**: TR(OPrSH)6-TC (*m*/*z* 796.4). Peaks **6** and **7** were confirmed by MS/MS; (**C**) HPLC of the purified TR6-TC from TR6-HET; (**D**) HPLC of the purified TR6-TC from TR6-HPT; (**E**) Mass spectrum of the purified TR6-TC from TR6-HET; (**F**) Mass spectrum of the purified TR6-TC from TR6-HPT Other side products are mentioned in Figures S3 and S4.

The kinetic behavior of five- and six-membered O-S acyl shifts was comparatively examined on the basis of disappearance rates of the starting materials. The model peptide handles, TR6-HET for five-membered acyl shift and TR6-HPT for six-membered acyl shift, were constructed, using the same peptide sequence (TIGGIR), to perceive only structural effect of C-terminal handle. It showed that TR6-HET was approximately twice as fast as TR6-HPT (Figure 1B), even though the product profiles from the two handles were only minimally different (Figure 2). Though it means that the five-membered acyl shift of HET handle is faster, it is more feasible to the production of side products than the six-membered acyl shift of HPT handle. Furthermore, a minute structural difference at the C-terminal residue between two peptides TR(r)6-HET with *l*- and *d*-Arg, showed that *l*-Arg was faster than *d*-Arg (Figure 1B). The C-terminal difference, however, only slightly affected the disappearance of the initial compound without changing the product pathway (Figure S5).

From product differentiation and kinetic studies of the two handles, two main problems could be explained: alkylation at the Arg site and racemization at the C-terminal in the acidic conditions as mentioned in a previous study [43]. The alkylation gave a reversible side product of the guanidino side chain of Arg from TR6-HET and Tr6-HET by ethylene sulfide, forming Tr(EtSH)6-TC and TR(EtSH)6-TC (peaks **6**, **7** in Figure 2A) while ca. 60% of TR(r)6-TC products was obtained (peaks **4** and **5** in Figure 2A). However, only 11% of TR6-HPT side products (peaks **9** and **10** in Figure 2B) were obtained from TR(r)6-TC products (peaks **6** and **7** in Figure 2B). It might be a reasonable explanation that the formation of propylene sulfide through the four-member ring intermediate of the HPT handle is substantially slower than that of the ethylene sulfide, through the three-member ring intermediate or, the released mercaptoethanol from the 1,2-elimination (**4** and **10**→**5** in Scheme 2).

Racemization of O-S acyl shift has been one of the major concerns in the conversion steps of the oxazolone pathway and peptide ligation [31,32,33,37,43]. Normally, the pathway (Scheme 3A) was established in a manner that the activated peptide esters are prone to racemization at the C-terminal residue via an oxazolone intermediate [37,44,45,46] even though this is unclear under some conditions [31,32]. However, a possible oxazolone pathway involving intermolecular interactions was not suitable to explain the racemization, since the conversion rate order was: racemization > O-S acyl shift > S-S exchange for TR6-HET and O-S acyl shift ≥ S-S exchange > racemization for TR6-HPT (Figures S3 and S4). Thus, it could not support the fact that no effect from other conditions except the different handle structure was there. The other rational argument against the oxazolone pathway was that Tr6-HET was detected at an early reaction time (peaks **3** and **5** in Figure S3, 5 min). This observation was not applicable to the oxazolone pathway because the released 2-mercaptoethanol has not to act as a nucleophile instead of thiocresol, which is in excess and appears as a better nucleophile in acid. Therefore, a plausible mechanism can be proposed for the C-terminal racemization on the basis of differentiation of the two handles that could play a dual role as both a nucleophile and base in the acidic conditions.

The proposed racemization mechanism is 7- and 8-membered ring interactions between the α-proton of C-terminal Arg, and thiol handle groups as a base (Scheme 3B). The hypothesis was that the α-proton should become more susceptible in an approaching motion of the thiol handles, which are proximal to the C-terminal carbonyl group and in *cis* configuration for O-S acyl shift. The situation would cause a fast enol formation in TR6-HET, but a much slower one in TR6-HPT, which is a more flexible handle. The proposed mechanism was well matched with Gellman’s study [47] where a restricted beta-peptide with *Z* configuration presented a strong NOE interaction in 6- and 7-membered rings. Further evidence of the 7-membered ring interaction has been established to give rise to a folding evidence, by the internal hydrogen bond in a small peptide-like structure [48].

## 3. Materials and Methods

### 3.1. Synthesis of Handles

All reagents and solvents were obtained from commercial suppliers and were used without further purification. Side-chain protected Fmoc amino acids used in the experiment were d-Arginine(2,2,4,6,7-pentamethyldihydrobenzofuran-5-sulfonyl) [d-Arg(Pbf)], Arginine(2,2,4,6,7-pentamethyldihydrobenzofuran-5-sulfonyl) [Arg(Pbf)], and Threonine(t-Buthyl) [Thr(tBu)]. Fmoc amino acids, 2-chlorotrityl chloride resin (1.2 mmol/g), (Benzotriazole-1-yloxyl) tris (dimethylamino) phosphonium hexafluoro phosphate [BOP], 2-mercaptoethanol, and 3-mercapto-1-propanol were purchased from Aldrich (Gyeonggi, Korea). Solvents used were purchased from SK chemicals (Gyeonggi, Korea).

#### Preparation of the HET and HPT Handles on 2-Chlorotrityl Chloride Resin and Peptide Synthesis

All peptides were synthesized manually. An amount of 1 g of 2-Chlorotrityl resin (1.2 mmol/g) was swollen in DMF for 2 h. The resin was washed with DMF and DCM. 2-Mercaptoethanol, ≥99.0% (Mw 78.13, d 1.114, 0.842 mL, ca. 12 mmol) and 3-mercapto-1-propanol, 95% (Mw 92.16, d 1.067, 1.1 mL, ca. 12 mmol) were added to DCM/DMF (*v/v* 1:1) solution of each reaction vessel with the resin. The reaction time was overnight for 2-Mercaptoethanol and 2 days for 3-mercapto-1-propanol to make the coupling complete. A triple coupling method was used, with a longer reaction time at each coupling to load the first amino acid on the resin. After chain assembly was completed, the peptides were deprotected and cleaved from the resins (250 mg, each) by treatment with reagent K (TFA/thioanisole/PhOH/H_2_O/EDT/TIS: 81.5/5/5/5/2.5/1) for 2 h for TR6-HET and TR6-HPT at room temperature. The crude peptides were precipitated with diethyl ether, dissolved in aqueous acetonitrile, and purified by preparative RP-HPLC on a Waters 600 HPLC module, using a C18 Phenomenex preparative column (250 mm × 10 mm, 10 µ). The yield of two handles was 70%–80%. The molecular weight of peptides was confirmed by 4800 MALDI TOF/TOF Analyzer from Applied Biosystems.

### 3.2. Tandem Switch Experiment for O-S Acyl Shift

The conversion of tandem switch for 5- and 6-membered O-S acyl shift was conducted in 2–3 mg peptides within 1 mL total volume including TFA, 0.05%–1.5% TfOH as a freshly pre-made solution of 10% TfOH/TFA by volume, and 5% thiocresol (50 µL) at room temperature for 2 h, 4 h, and more. The remaining thiocresol was removed by ether precipitation twice. The yield of all peptide-TC products was obtained by analytical RP-HPLC purification on a Shimadzu 10 series module with Diode Array UV detection, using a Vydac C18 column, with a linear gradient of 2% buffer B to 100% buffer B for 40 min and retained for 10 min in 100% buffer B after 40 min (Buffer A = 0.05% TFA in water; buffer B = 0.045% TFA in 60% acetonitrile in water). However, the area percentage of HPLC for other side products of small amounts such as “before 22 min” and “after 34 min” was employed (Supplementary Materials Table S1). The molecular weight of all products was confirmed by 4800 MALDI TOF/TOF Analyzer from Applied Biosystems.

## 4. Conclusions

In summary, the O-S acyl shift of peptide-HET and peptide-HPT was dependent on acidity and the protonation state of the ester carbonyl. Addition of a catalytic TfOH accelerated the O-S acyl shift to afford a thioester. The addition of an external thiol into the reaction mixture permitted the second “thiol switch” reaction and favored the formation of stable thioester 6 (peak **5** in Figure 2) by terminating the equilibration of the acyl migrations. The design of the handle difference provides a new finding that the thiol groups can be used as a base in the acidic conditions. The C-terminal HPT handle for O-S acyl shift could be a more promising structure to reduce alkylation and racemization even though both handles were manipulated from convenient starting materials which are simple and feasible to provide thioester peptides compatible with Fmoc chemistry. However, the acidic effect on structures of other C-terminal amino acids and longer peptide sequences will be further studied to establish a relationship between O-S acyl shift and side products.

## Supplementary Materials

Supplementary materials can be accessed at: http://www.mdpi.com/1420-3049/21/11/1599/s1.
