# Supplementary material for: The C-Terminal O-S Acyl Shift Pathway under Acidic Condition to Propose Peptide-Thioesters
Source: Molecules. 2016 Nov 17;21(11):1559. doi: 10.3390/molecules21111559 (PMC6272864; doi:10.3390/molecules21111559)
Supplement: Supplementary file 1 [file molecules-21-01559-s001.pdf]

Supplementary Materials: The C-Terminal O-S Acyl Shift Pathway under Acidic Condition to Propose Peptide-Thioesters

Bo Mi Kim

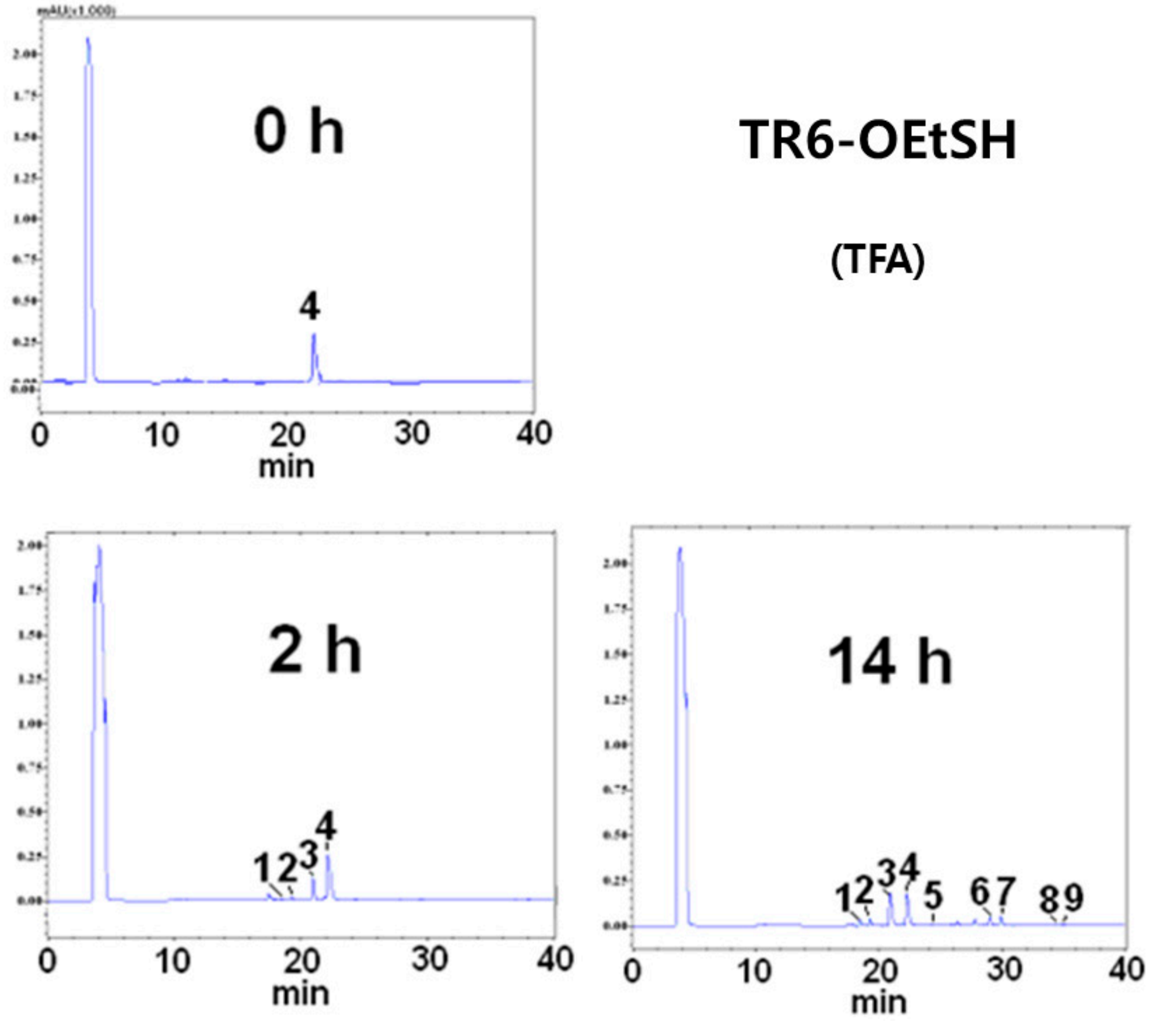

1: Tr6-SEtOH (*m*/*z* 676.1) **2**: TR6-SEtOH (*m*/*z* 676.1) **3**: Tr6-OEtSH (*m*/*z* 676.1) which was confirmed by direct synthesis **4**: TR6-OEtSH (*m*/*z* 676.1) **5**: S-S dimer of TR6-HET (*m*/*z* 1349.9). The CF_3CO-adducted positions from TIGGIR-HET were not confirmed but we assumed **6** as Tr($COCF_3$)6-HET (*m*/*z* 818.1), **7** as Tr($COCF_3$)6-HET (*m*/*z* 818.1), **8** as (CF_3CO)Tr6-HET (*m*/*z* 818.1), and **9** as (CF_3CO)TR6-HET (*m*/*z* 818.1).

Figure S1. The product profile of TR6-OEtSH in TFA at 0 h, 2 h and 14 h.

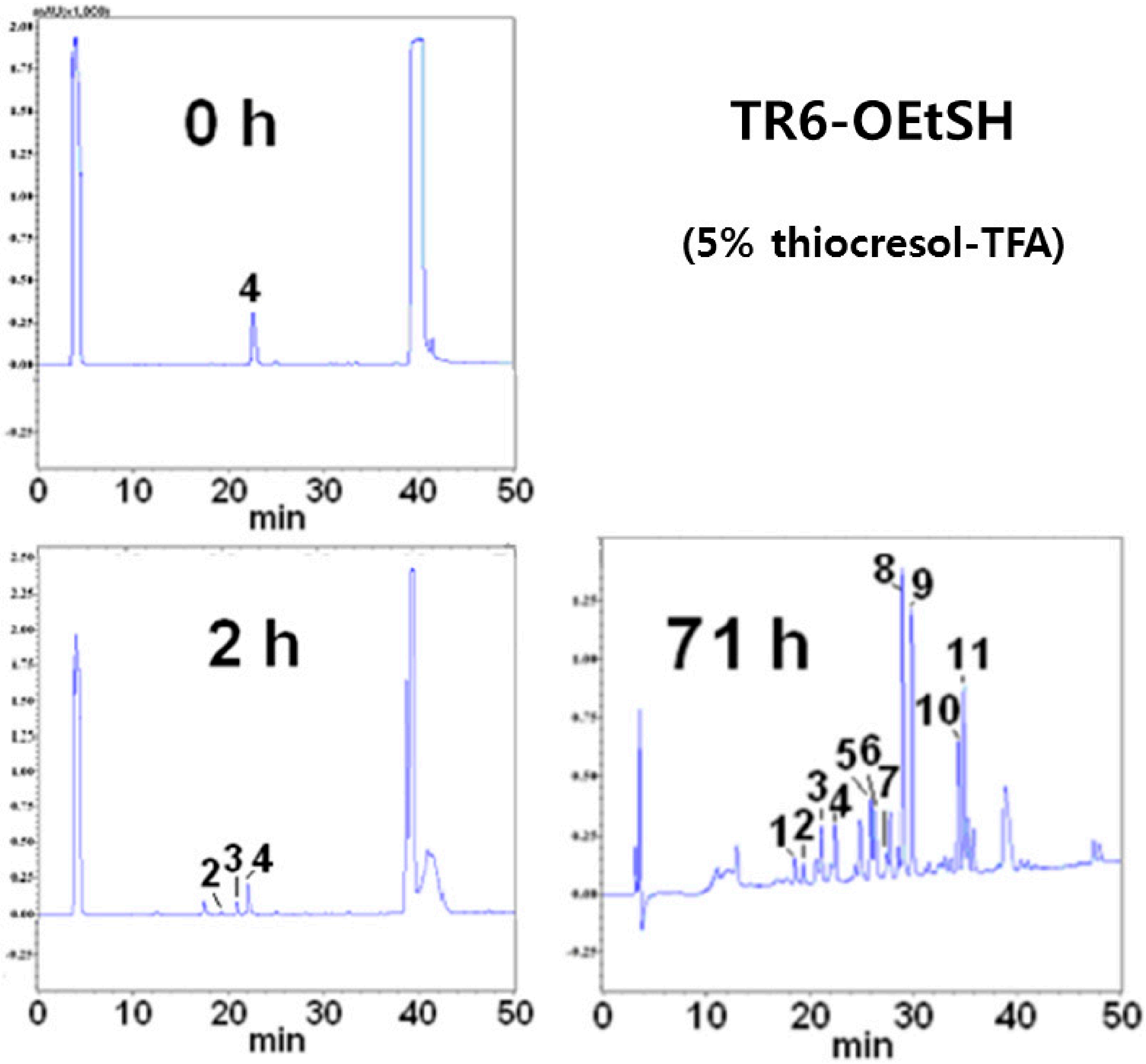

1: Tr6-SEtOH (*m/z* 676.1) **2**: TR6-SEtOH (*m/z* 676.1) **3**: Tr6-OEtSH (*m/z* 676.1) which was confirmed by direct synthesis **4**: TR6-OEtSH (*m/z* 676.1) **7**: Tr6-TC (*m/z* 722.1) The CF_3CO-adducted positions from TIGGIR-HET were not confirmed but we assumed **5** as Tr6-OEtS-$COCF_3$ (*m/z* 818.1), **6** as TR6-OEtS-$COCF_3$ (*m/z* 818.1), **8** as Tr($COCF_3$)6-HET (*m/z* 818.1), **9** as Tr($COCF_3$)6-HET (*m/z* 818.1), **10** as (CF_3CO) Tr6-HET (*m/z* 818.1), and **11** as (CF_3CO)TR6-HET (*m/z* 818.1). The TR6-TC was not detected because of interference of a CF_3CO-adducted product.

Figure S2. The product profile of TR6-OEtSH in 5%TfOH-TFA at 0 h, 2 h and 71 h.

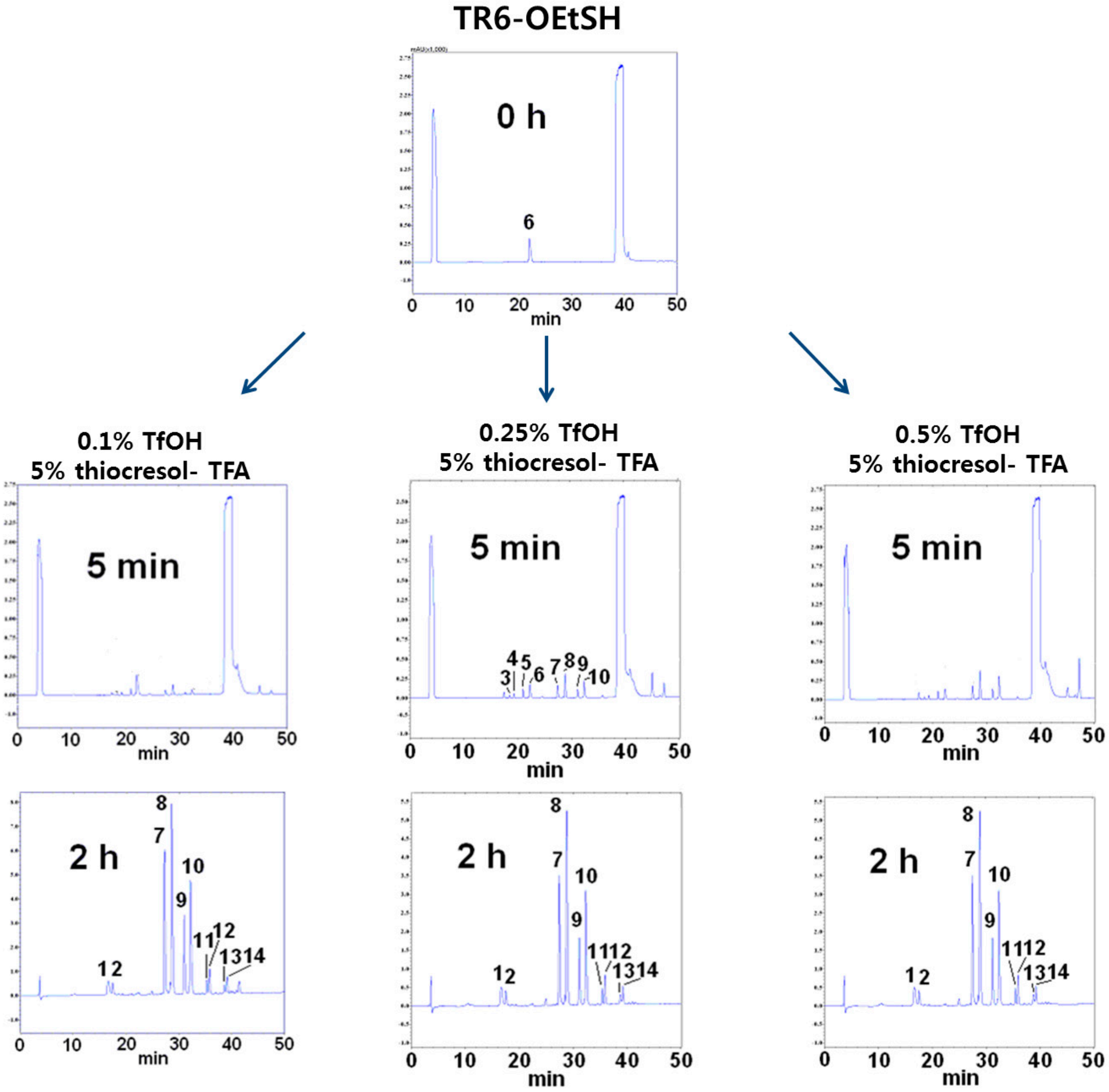

1, **2**: Tr6-OH & TR6-OH (*m*/*z* 616.4) **3**: Tr6-SEtOH (*m*/*z* 676.4) **4**: TR6-SEtOH (*m*/*z* 676.4) **5**: Tr6-OEtSH (*m*/*z* 676.4) which was confirmed by direct synthesis **6**: TR6-OEtSH (*m*/*z* 676.4) **7**: Tr6-TC (*m*/*z* 722.4) **8**: TR6-TC (*m*/*z* 722.4); **9**: Tr(EtSH)6-TC (*m*/*z* 782.4) **10**: TR(EtSH)6-TC (*m*/*z* 782.4) The CF_3CO-adducted positions of TIGGIR-TC were not confirmed but we assumed **11** as (CF_3CO)Tr6-TC (*m*/*z* 818.4), **12** as (CF_3CO)TR6-TC (*m*/*z* 818.4), **13** as (CF_3CO)Tr(EtSH)6-TC (*m*/*z* 878.4), **14** as (CF_3CO)TR(EtSH)6-TC (*m*/*z* 878.4).

Figure S3. The product profile of TR6-OEtSH in 0.1%TfOH-5%TC-TFA, 0.25%TfOH-5%TC-TFA and 0.5%TfOH-5%TC-TFA at 0 h, 5 min and 2 h.

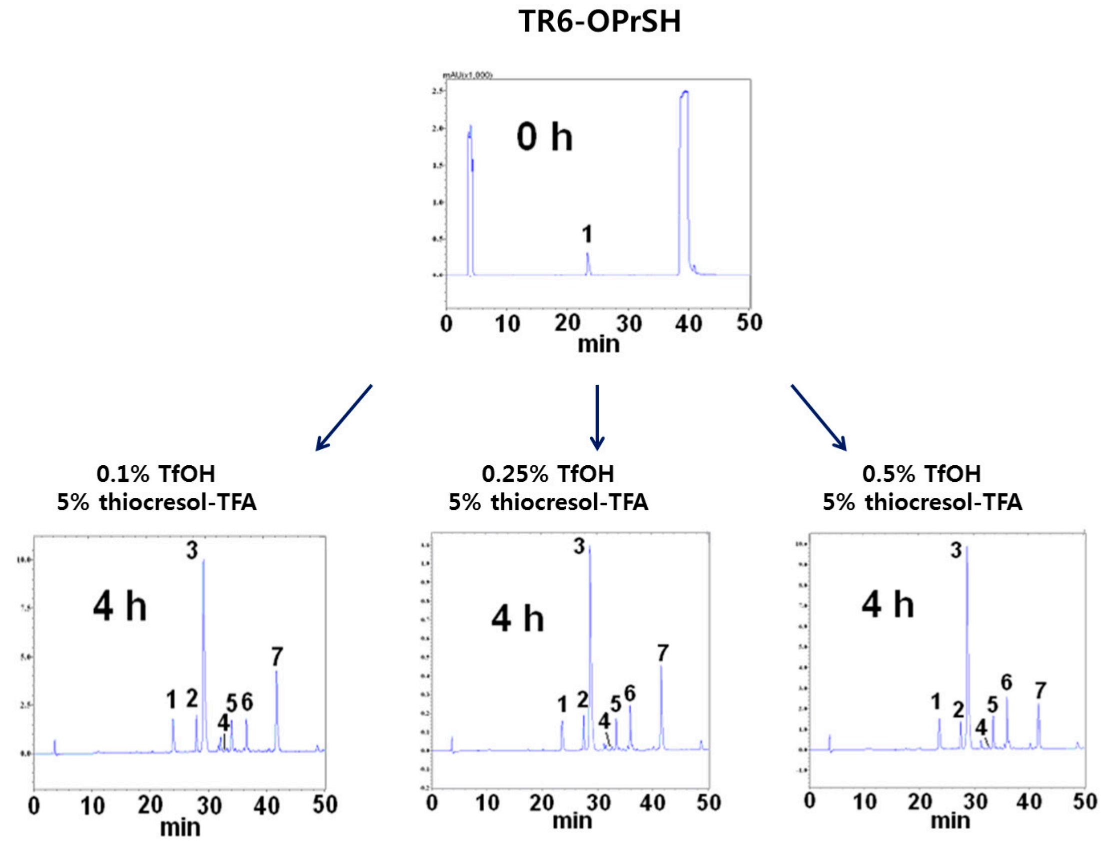

1: TR6-OPrSH (*m*/*z* 690.3) **2**: Tr6-TC (*m*/*z* 722.4) **3**: TR6-TC (*m*/*z* 722.4) **4**: Tr(PrSH)6-TC (*m*/*z* 796.3) **5**: TR(PrSH)6-TC (*m*/*z* 796.3) The CF_3CO-adduct position of TIGGIR-TC was not confirmed but we assumed **6** as (CF3CO) TR6-TC (*m*/*z* 818.3). **7**: unknown (*m*/*z* 1016.4)

Figure S4. The product profile of TR6-OPrSH in 0.1%TfOH-5%TC-TFA, 0.25%TfOH-5%TC-TFA and 0.5%TfOH-5%TC-TFA at 0 h and 4 h

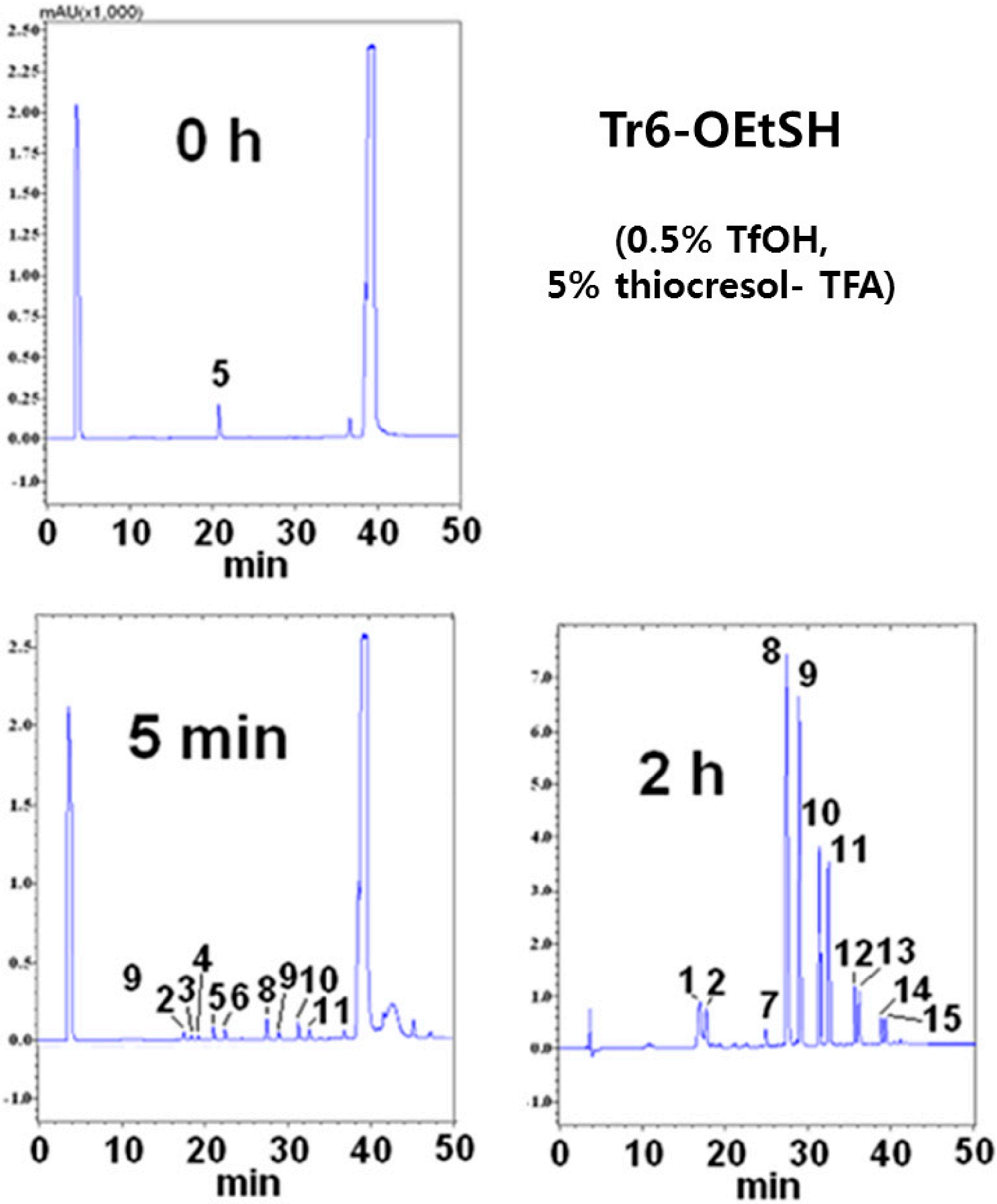

1, 2: Tr6-OH & TR6-OH (*m*/*z* 616.4) **3**: Tr6-SEtOH (*m*/*z* 676.4) **4**: TR6-SEtOH (*m*/*z* 676.5) **5**: Tr6-OEtSH (*m*/*z* 676.4) **6**: TR6-OEtSH (*m*/*z* 676.5) **7**: (CF_3CO)Tr6-OH (*m*/*z* 712.4) **8**: Tr6-TC (*m*/*z* 722.4) **9**: TR6-TC (*m*/*z* 722.5) **10**: Tr(EtSH)6-TC (*m*/*z* 782.5) **11**: TR(EtSH)6-TC (*m*/*z* 782.4) The CF_3CO-adducted positions of TIGGIR-TC were not confirmed but we assumed **12** as (CF_3CO)Tr6-TC (*m*/*z* 818.4), **13** as (CF_3CO)TR6-TC (*m*/*z* 818.4), **14** as (CF_3CO)Tr(EtSH)6-TC (*m*/*z* 878.6), **15** as (CF_3CO)TR(EtSH)6-TC (*m*/*z* 878.4).

Figure S5. The product profile of Tr6-OEtSH in 0.5%TfOH-5%TC-TFA at 0 h, 5 min, and 2 h.

Table S1. The product profile of TIGGIR-HET and TIGGIR-HPT in **X**% TfOH-5%TC-TFA.

**TIGGIR-OEtSH (TR6-HET)**								
**X (%) (TfOH)**	**Work-Up Time (h)**	**Remaining Starting (%)** [b]	**TR6- & Tr6-TC (%)** [c]	**TR6-TC Ratio (*l*/*d*)**	**TR6- & Tr6-TC+60 (%)** [c]	**TR6-TC+60 Ratio (*l*/*d*)**	**before 22 min (%)** [d]	**after 34 min (%)** [d]
0.05	2	4.1	42.0	1.4	22.1	1.3	6.4	23.4
0.10	2	-	49.3	1.5	25.9	1.6	4.1	20.8
0.20	2	-	52.2	1.5	26.6	1.7	5.3	14.4
0.30	2	-	52.1	1.5	27.1	1.8	5.8	13.7
0.40	2	-	52.2	1.7	27.4	1.9	5.4	13.4
0.50	2	-	49.1	1.7	29.7	2.0	6.8	13.0
1.00 [a]	2	-	39.3	1.9	28.6	2.2	9.4	13.5
1.50 [a]	2	-	33.2	2.0	29.5	2.3	14.5	10.4
**TIGGIR-OPrSH (TR6-HPT)**								
**X (%) (TfOH)**	**Work-Up Time (h)**	**Remaining Starting (%)** [b]	**TR6- & Tr6-TC (%)** [c]	**TR6-TC Ratio (*l*/*d*)**	**TR6- & Tr6-TC+74 (%)** [c]	**TR6-TC+74 Ratio (*l*/*d*)**	**before 22 min (%)** [d]	**after 34 min (%)** [d]
0.13	10.3	4.6	60.6	6.9	6.2	4.2	0.9	25.4
0.28	7.2	3.8	59.9	9.9	5.8	4.8	0.2	28.1
0.33	6	6.9	57.7	10.3	5.8	6.3	0.4	25.6
0.55	4	6.6	59.9	12.9	6.5	12.0	0.6	24.0
1.00 [a]	4	9.9	56.3	15.2	6.6	17.3	1.2	17.2

[a] small portions (5%–15%) of the total amount were decreased in comparison of those of the lower TfOHs. [b] a relative percentage from the starting material, [c] isolated yield, [d] a relative percentage of total integration.

Peptide Sequence	Retention Time (min) *	Expected Mass (*m*/*z*)	Observed Mass (*m*/*z*)
TIGGIR-OEt-SH (TR6-HET, Scheme 2, **1**)	22.1	675.7	676.3
TIGGIr-OEt-SH (Tr6-HET, SI Figure 6, **5**)	20.7	675.7	676.4
TIGGIR-thiocresol (TR6-TC, Scheme2, **6**)	28.5	721.8	722.4
TIGGIr-thiocresol (Tr6-TC, Figure 2A, **4**)	27.1	721.8	722.3
TIGGIr(CH_2CH_2SH)-thiocresol (Figure 2A, **6**)	30.9	781.9	782.5
TIGGIR(CH_2CH_2SH)-thiocresol (Scheme 2, **8**)	32.1	781.9	782.4
TIGGIR-OPr-SH (TR6-HPT, Figure 2B, **8**)	23.3	689.7	690.8
TIGGIr($CH_2CH_2CH_2SH$)-thiocresol (Figure 2B, **9**)	32.3	795.9	796.8
TIGGIR($CH_2CH_2CH_2SH$)-thiocresol (Figure 2B, **10**)	33.4	795.9	796.8

* HPLC condition: 2% buffer B to 100% buffer B at 40 min (buffer A: H_2O with 0.05% TFA, buffer B: 60% CH_3CN/H_2O with 0.045% TFA).

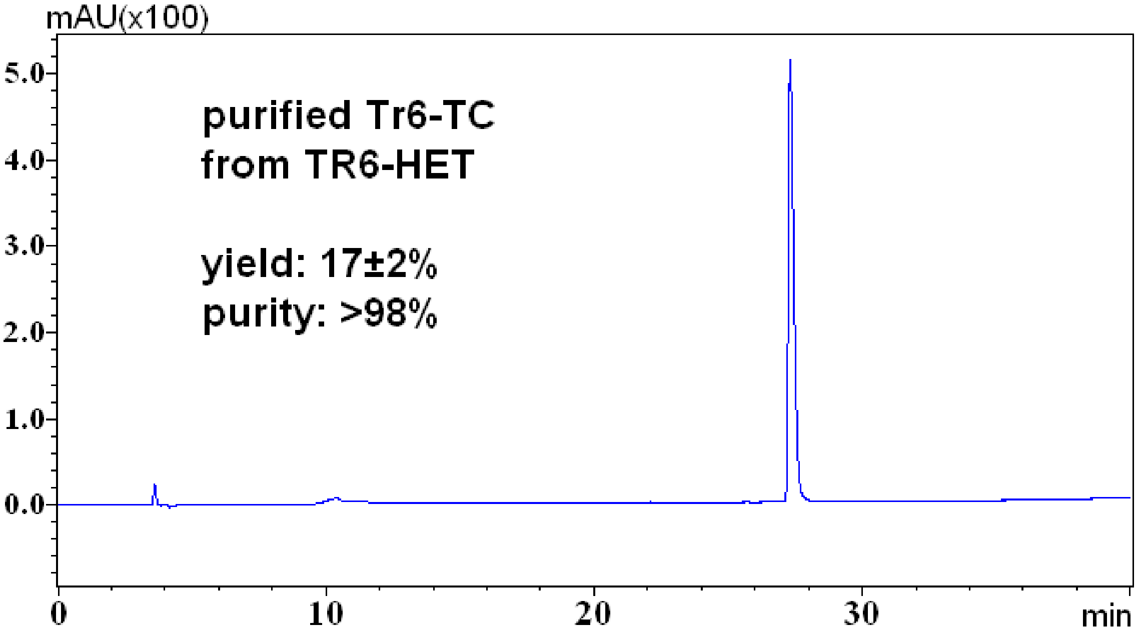

Purified HPLC of TIGGIr-TC (Tr6-TC) after the TR6-OEtSH reaction

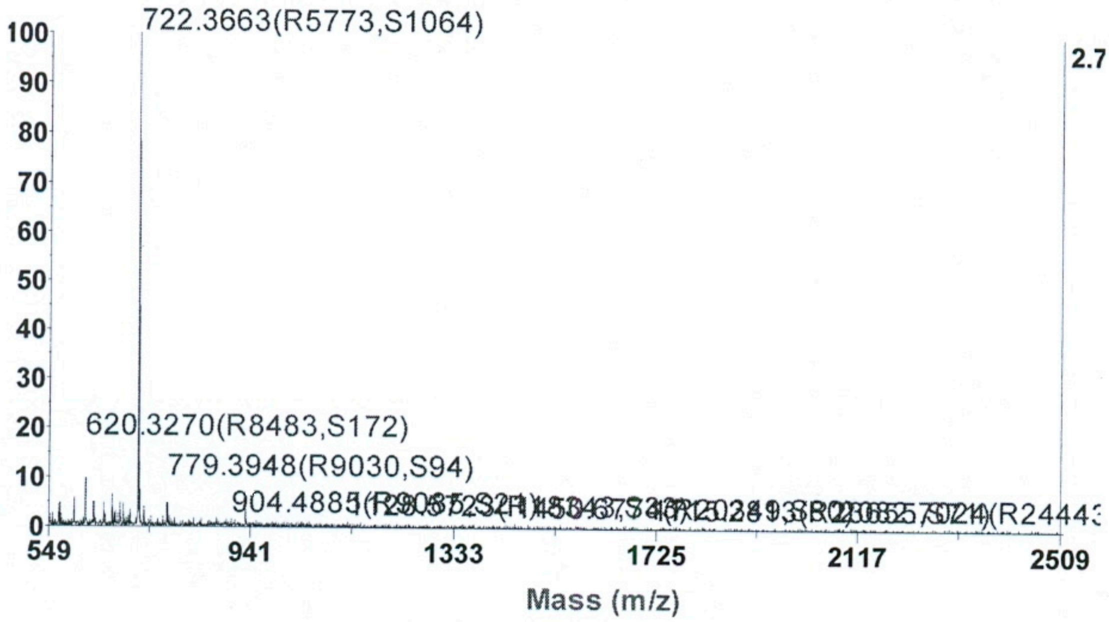

Mass Spectrum of Tr6-TC

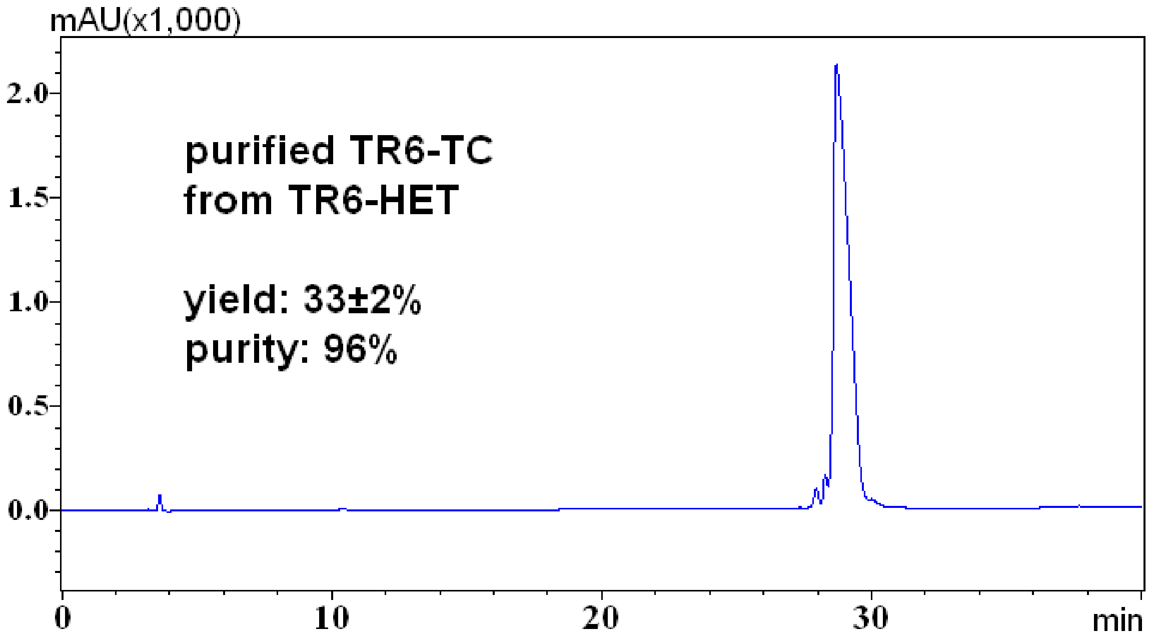

Purified HPLC of TIGGIR-TC (TR6-TC) after the TR6-OEtSH reaction

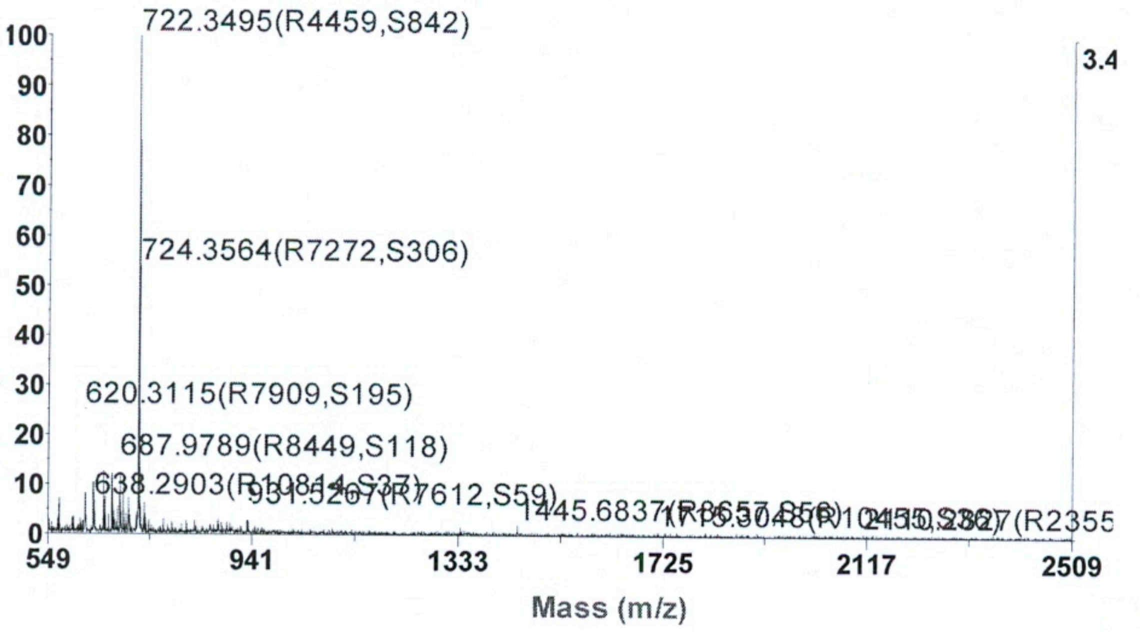

Mass Spectrum of TR6-TC

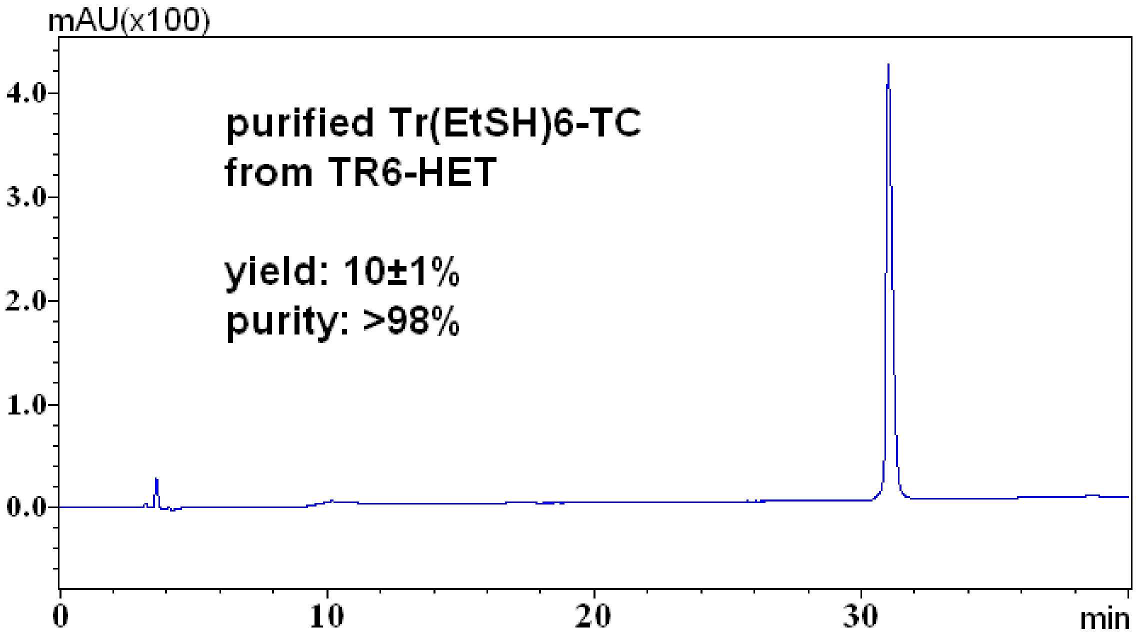

Purified HPLC of TIGGIr(EtSH)-TC [Tr(EtSH)6-TC] after the TR6-OEtSH reaction

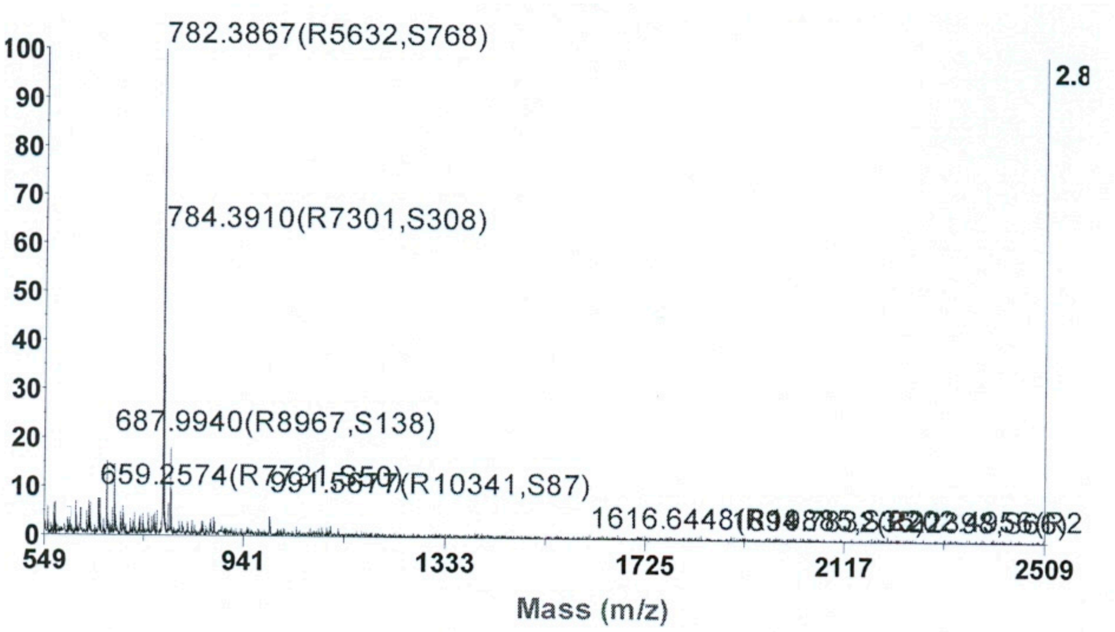

Mass Spectrum of Tr(EtSH)6-TC

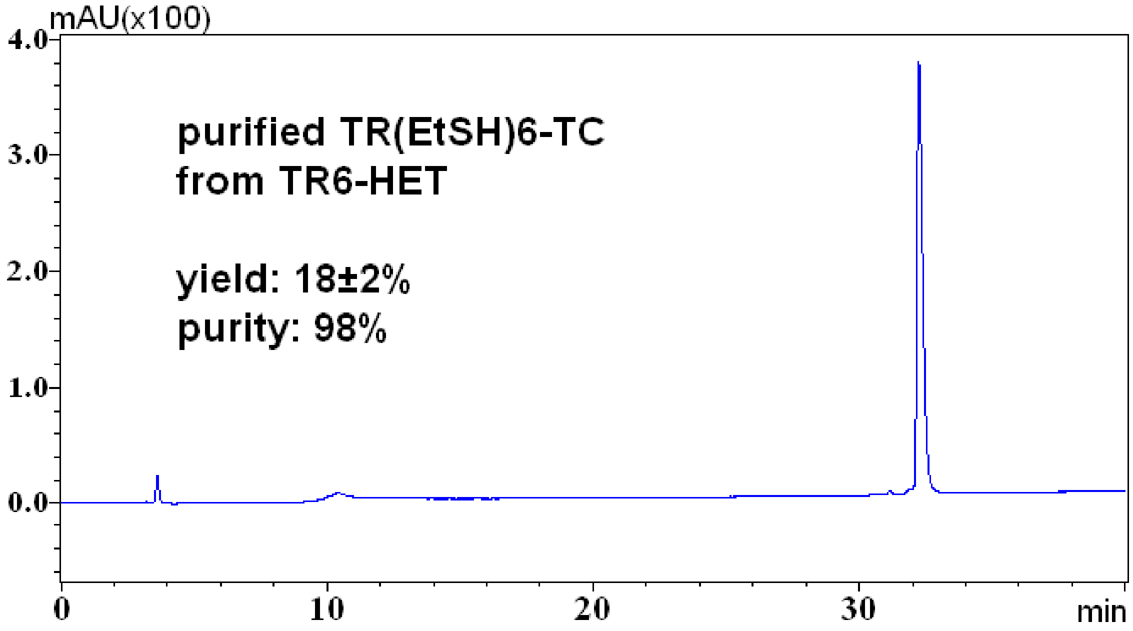

Purified HPLC of TIGGIR(EtSH)-TC [TR(EtSH)6-TC] after the TR6-OEtSH reaction

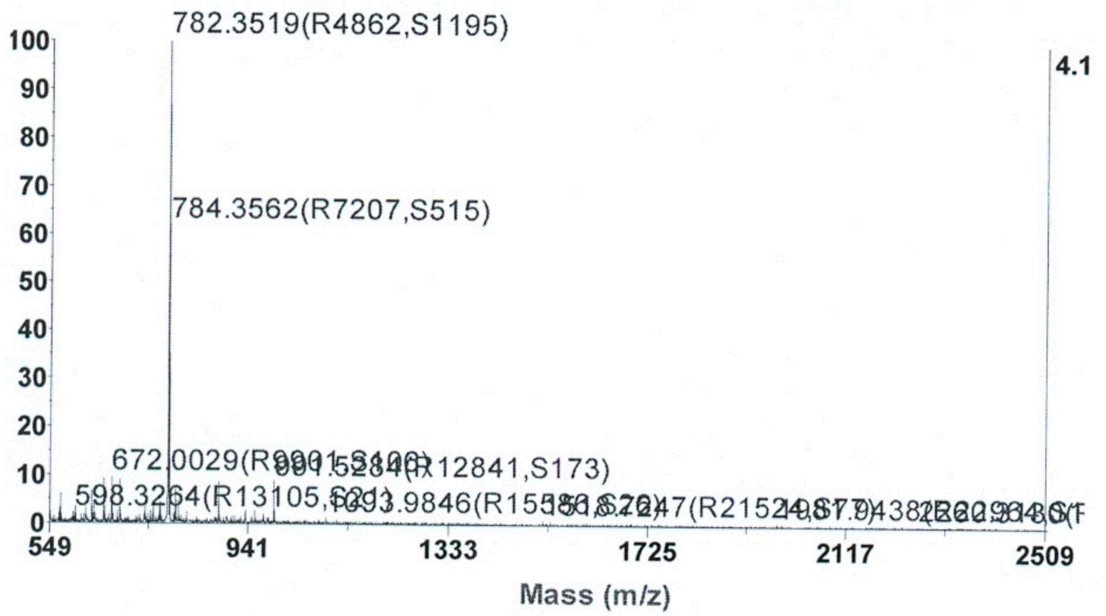

Mass Spectrum of TR(EtSH)6-TC

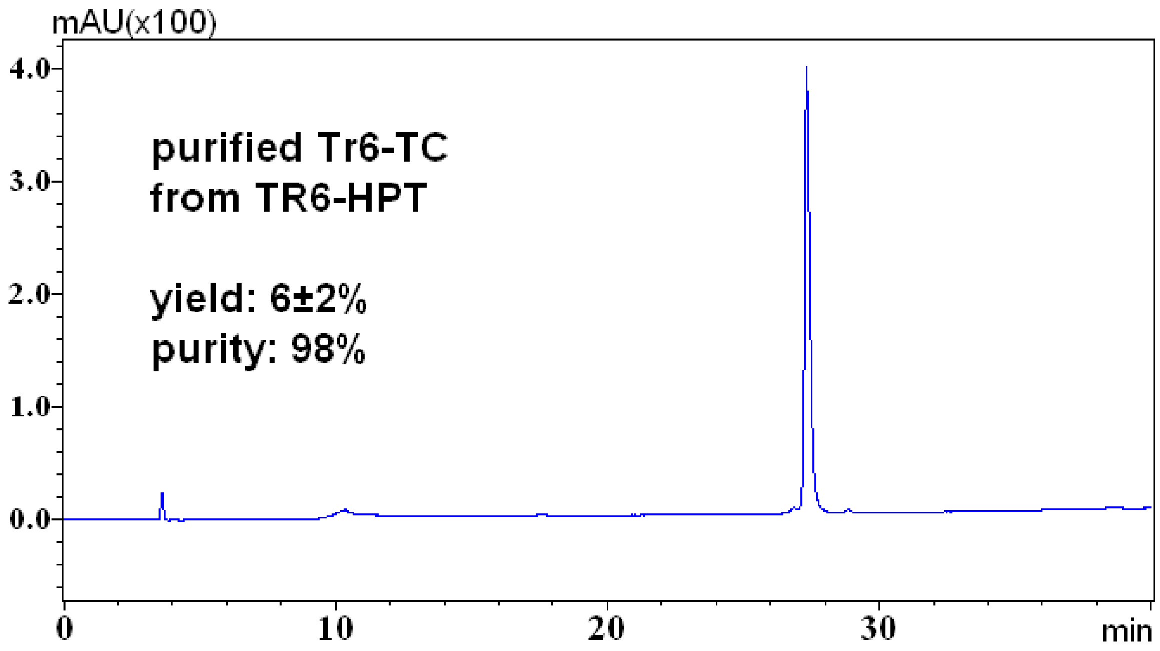

Purified HPLC of TIGGIr-TC (Tr6-TC) after the TR6-OPrSH reaction

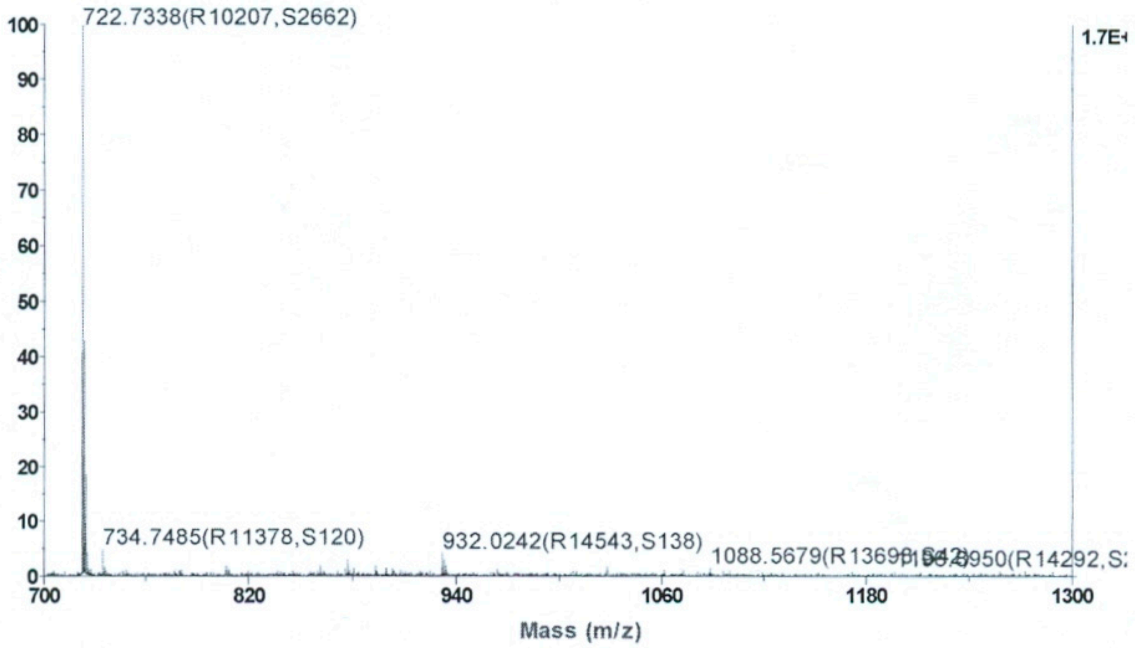

Mass Spectrum of Tr6-TC

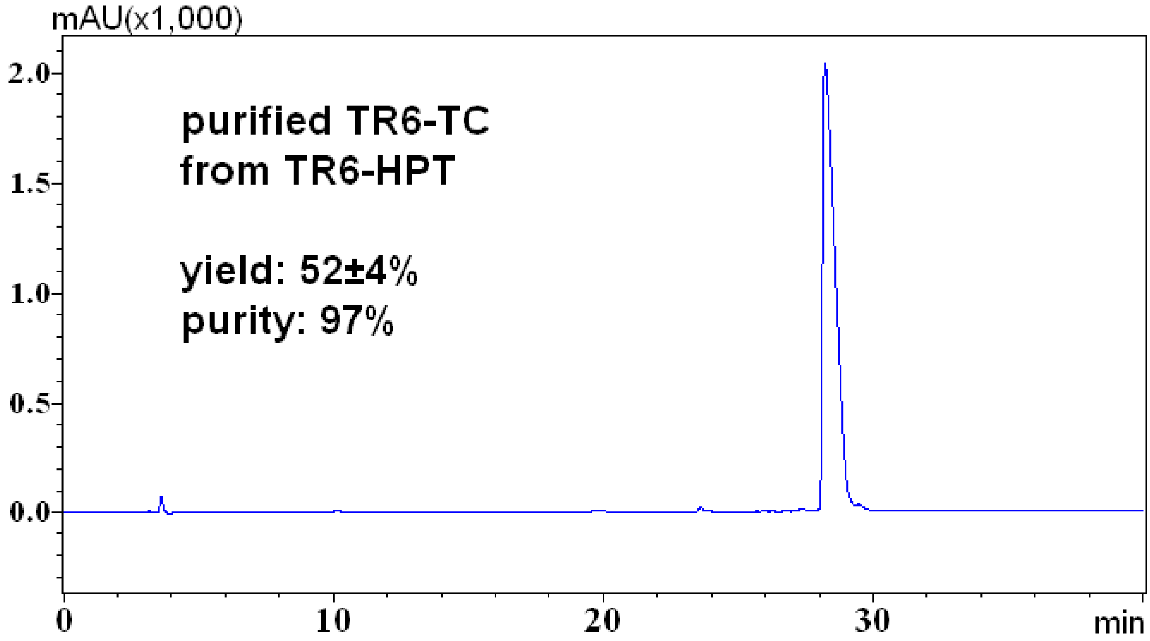

Purified HPLC of TIGGIR-TC (TR6-TC) after the TR6-OPrSH reaction

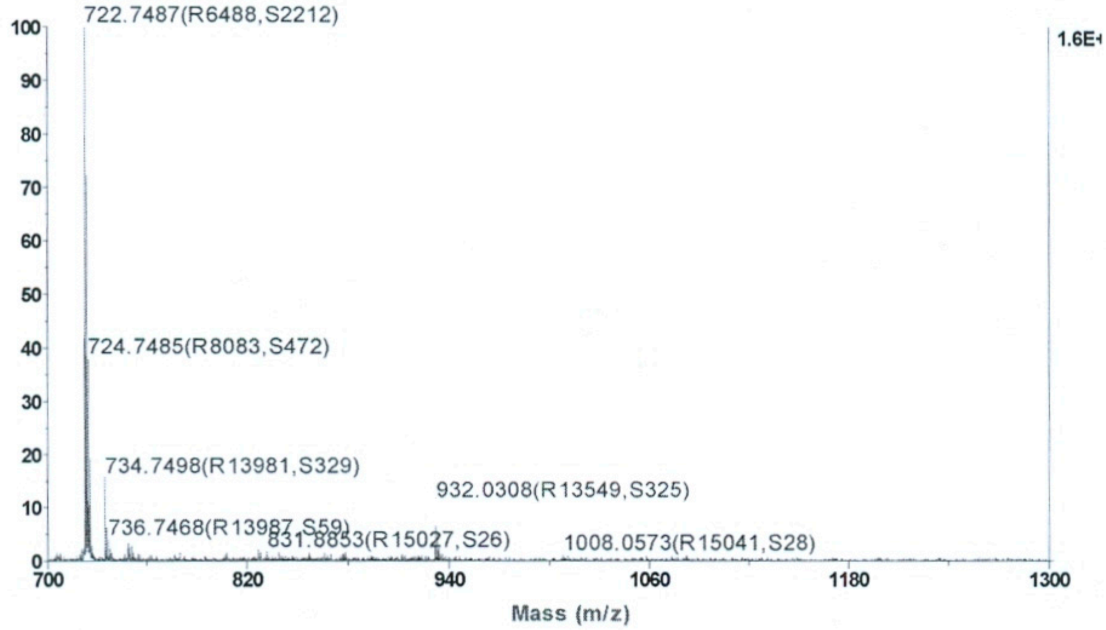

Mass Spectrum of TR6-TC

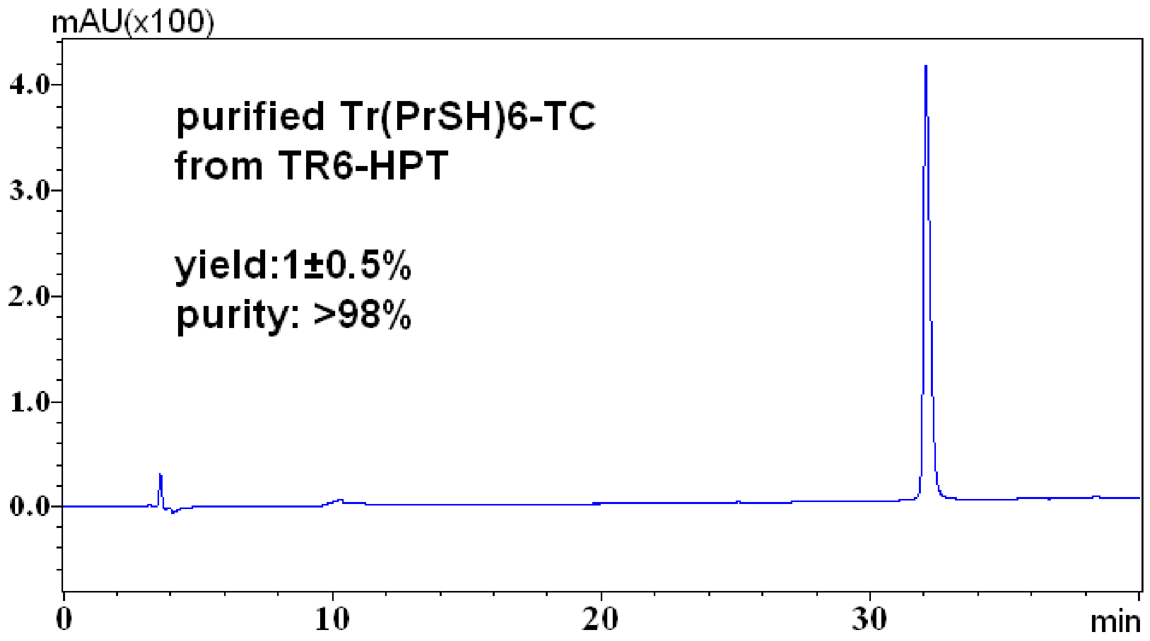

Purified HPLC of TIGGIr(PrSH)-TC [Tr(PrSH)6-TC] after the TR6-OPrSH reaction

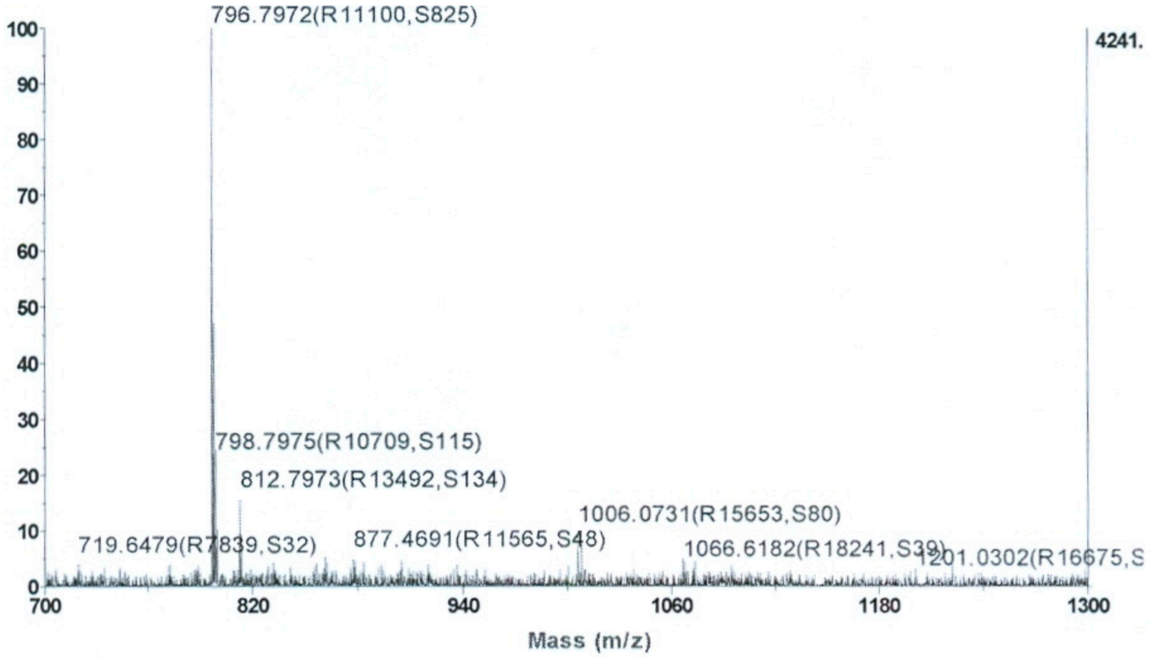

Mass Spectrum of Tr(PrSH)6-TC

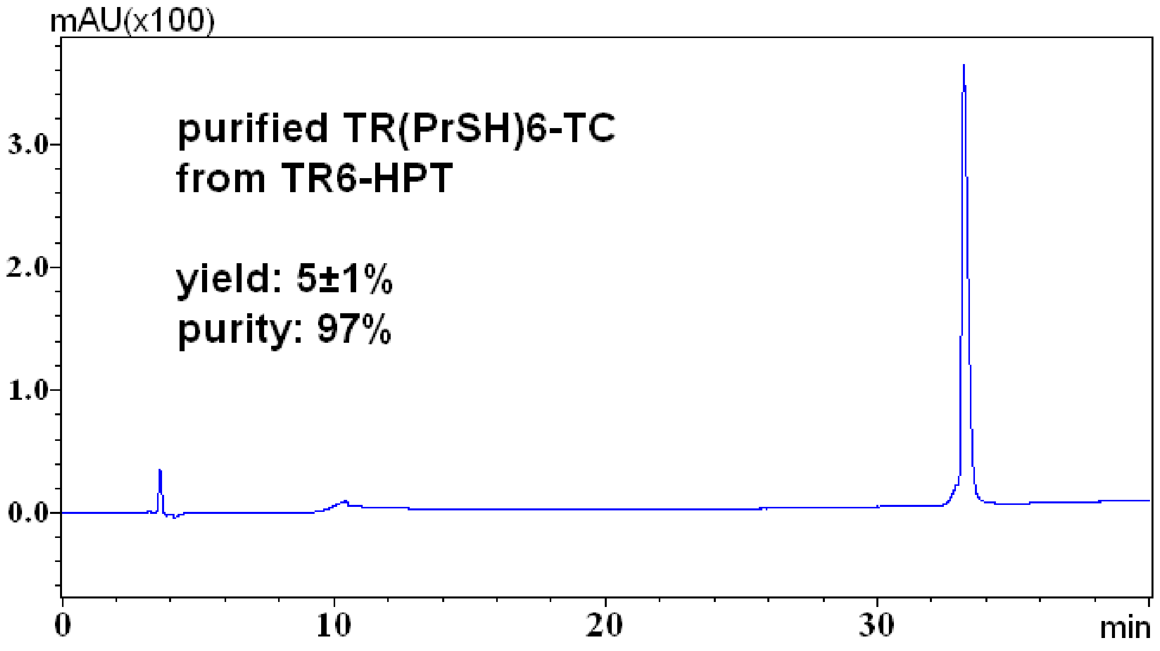

Purified HPLC of TIGGIR(PrSH)-TC [TR(PrSH)6-TC] after the TR6-OPrSH reaction

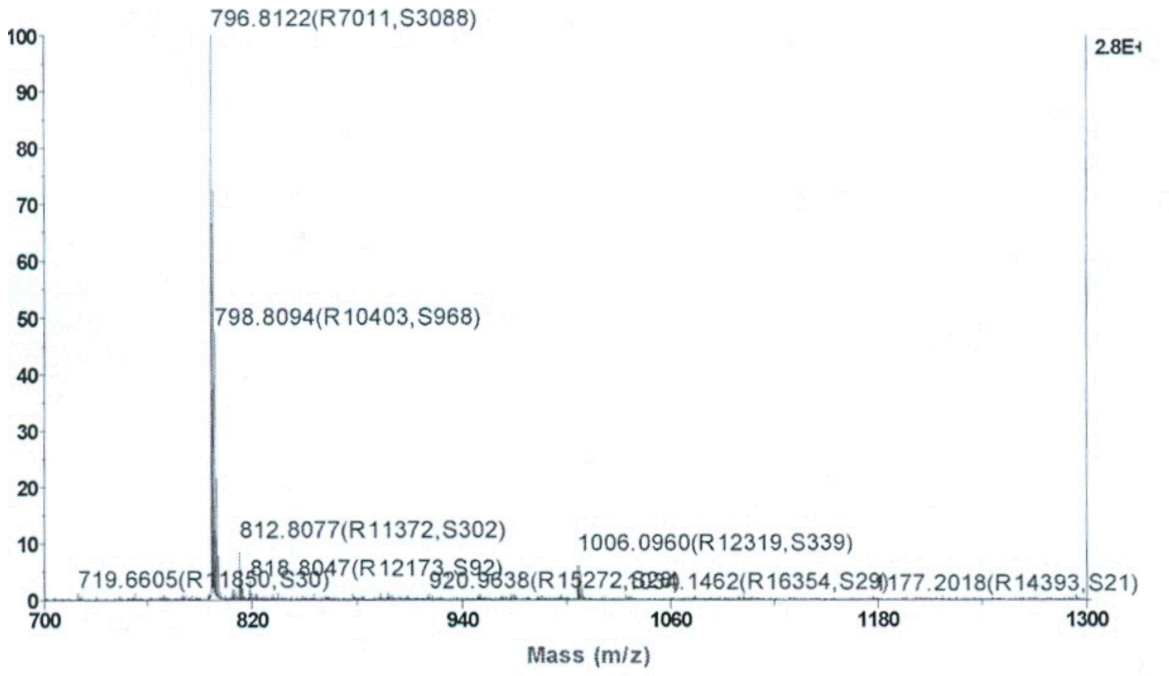

Mass Spectrum of TR(PrSH)6-TC
